# Sample-Index Misassignment Impacts Tumour Exome Sequencing

**DOI:** 10.1038/s41598-018-23563-4

**Published:** 2018-03-28

**Authors:** Daniel Vodák, Susanne Lorenz, Sigve Nakken, Lars Birger Aasheim, Harald Holte, Baoyan Bai, Ola Myklebost, Leonardo A. Meza-Zepeda, Eivind Hovig

**Affiliations:** 1 Institute of Clinical Medicine, Faculty of Medicine, University of Oslo, Oslo, Norway; 2 Norwegian Cancer Genomics Consortium, Institute for Cancer Research, The Norwegian Radium Hospital/Oslo University Hospital, Oslo, Norway; 3 Department of Tumor Biology, Institute for Cancer Research, The Norwegian Radium Hospital/Oslo University Hospital, Oslo, Norway; 4 Department of Core Facilities, Institute for Cancer Research, The Norwegian Radium Hospital/Oslo University Hospital, Oslo, Norway; 5 Department of Oncology, Cancer Clinic, Oslo University Hospital, Oslo, Norway; 6 Centre for Cancer Biomedicine, University of Oslo, Oslo, Norway; 7 Department of Cancer Immunology, Institute for Cancer Research, Oslo University Hospital, Oslo, Norway; 8 Department of Clinical Science, University of Bergen, Bergen, Norway; 9 Genomics Core Facility, Department of Core Facilities, Institute for Cancer Research, The Norwegian Radium Hospital/Oslo University Hospital, Oslo, Norway; 10 Department of Cancer Genetics and Informatics, Oslo University Hospital, Oslo, Norway; 11 Department of Informatics, University of Oslo, Oslo, Norway

## Abstract

Sample pooling enabled by dedicated indexes is a common strategy for cost-effective and robust high-throughput sequencing. Index misassignment leading to mutual contamination between pooled samples has however been described as a general problem of the latest Illumina sequencing instruments utilizing exclusion amplification. Using real-life data from multiple tumour sequencing projects, we demonstrate that index misassignment can induce artefactual variant calls closely resembling true, high-quality somatic variants. These artefactual calls potentially impact cancer applications utilizing low allelic frequencies, such as in clonal analysis of tumours. We discuss the available countermeasures with an emphasis on improved library indexing methods, and provide software that can assist in the identification of variants that may be consequences of index misassignment.

## Introduction

Identification of somatic variants by next-generation sequencing has become an important technique in cancer research by pinpointing the genomic causes of tumour phenotypes. An increasing number of examples have further shown that genomic aberrations have prognostic value and can inform rational clinical deployment of targeted cancer drugs^1,2^. As of now, next-generation sequencing technologies enable affordable assaying of variation in the entire tumour genome within days, but despite the availability of specialized software tools, somatic variant calling continues to pose challenges. Notably, the inherent complexity of tumours, as exemplified by aneuploidy, tumour heterogeneity, and sample impurity, often leads to important somatic variants only being detectable in low allelic fractions (AFs)^3–7^. AFs can be further affected due to technical reasons, such as the inability to accurately represent allelic ratios at genomic loci with low coverage^8^. As a consequence of the necessarily high required sensitivity, somatic variant calling is susceptible to random noise, systematic artefacts, and sample contamination^9,10^. When unnoticed, false positive variant calls can contribute to high costs incurred by follow-up analyses and experiments, and in the worst case support inadequate therapeutic strategies.

As previously described in the context of RNA-seq applications^11^, when material from multiple samples is being pooled together before sequencing, the sequencing technology itself can be a source of noticeable sample cross-contamination. Sample pooling (also called sample multiplexing) is a standard means of dividing the throughput of a sequencing instrument among multiple samples, relying on index sequences that uniquely barcode the material of each involved sample. Sample index misassignment, a phenomenon most evident on Illumina instruments utilizing exclusion amplification chemistry (ExAmp) and patterned flow cell technology (i.e., HiSeq 3000/4000/X Ten and NovaSeq), effectively leads to transfer of individual sequencing reads between samples included into a common pool^11^.

Our analysis of high-coverage tumour sequencing data shows that index misassignment is a source of false positive somatic variant calls in a form of true variation obtained from co-multiplexed samples.

## Results

We investigated tumour-normal sample pairs of three different tumour types: diffuse large B-cell lymphoma, follicular lymphoma, and sarcoma. All samples were collected from cancer patients in Norway, and deep exome sequencing (median coverages: 315X for tumour samples, 146X for controls) was carried out on local Illumina instruments: three HiSeq 2000/2500 instruments utilizing bridge amplification (generating 42 diffuse large B-cell lymphoma samples and 31 sarcoma samples) and a HiSeq 4000 employing ExAmp (generating 81 follicular lymphoma samples and 77 sarcoma samples). All samples were subject to standardized library preparation, sequencing, and bioinformatics analysis, as adopted by the Norwegian Cancer Genomics Consortium (NCGC, http://cancergenomics.no) (Methods, Supplementary Information).

In our assessment of index misassignment, we have examined the consequences for calling of somatic single nucleotide variants (SSNVs). For increased reliability, all tests and analyses were limited to SSNVs agreed upon by two independent variant callers (MuTect and Strelka)^3,12^. Sample-wise contamination estimates, our primary measure of contamination, were generated by Conpair^13^, based on the allelic composition on several thousand genomic marker sites in a given matched sample pair. Our tests on simulated data suggest that Conpair provides consistent quantifications, despite apparent progressive underestimation dependent on the number of contributing contamination sources (Supplementary Tables 1 and 2, Supplementary Information). We attribute this underestimation to decreasing contaminant variant AFs in sample pools of increasing size (Supplementary Fig. 1) paired with the fact that Conpair’s contamination model is intended for two-sample mixtures only.

**Figure 1:**
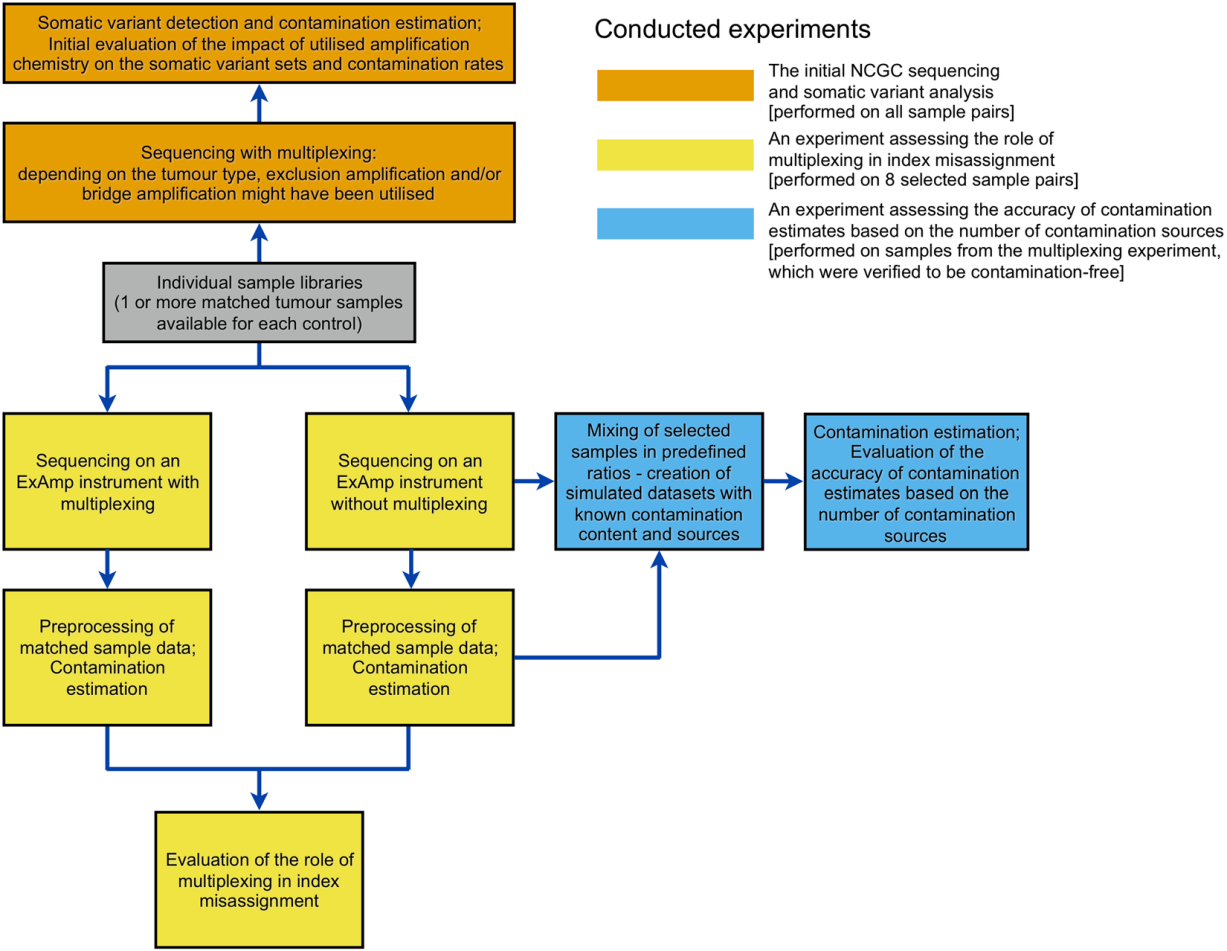
A schematic overview of the three main conducted experiments. The same library material was used for any given sample included in multiple experiments.

A schematic overview of all conducted experiments and their data dependencies is available on Fig. 1.

### Contamination rates

**Figure 2:**
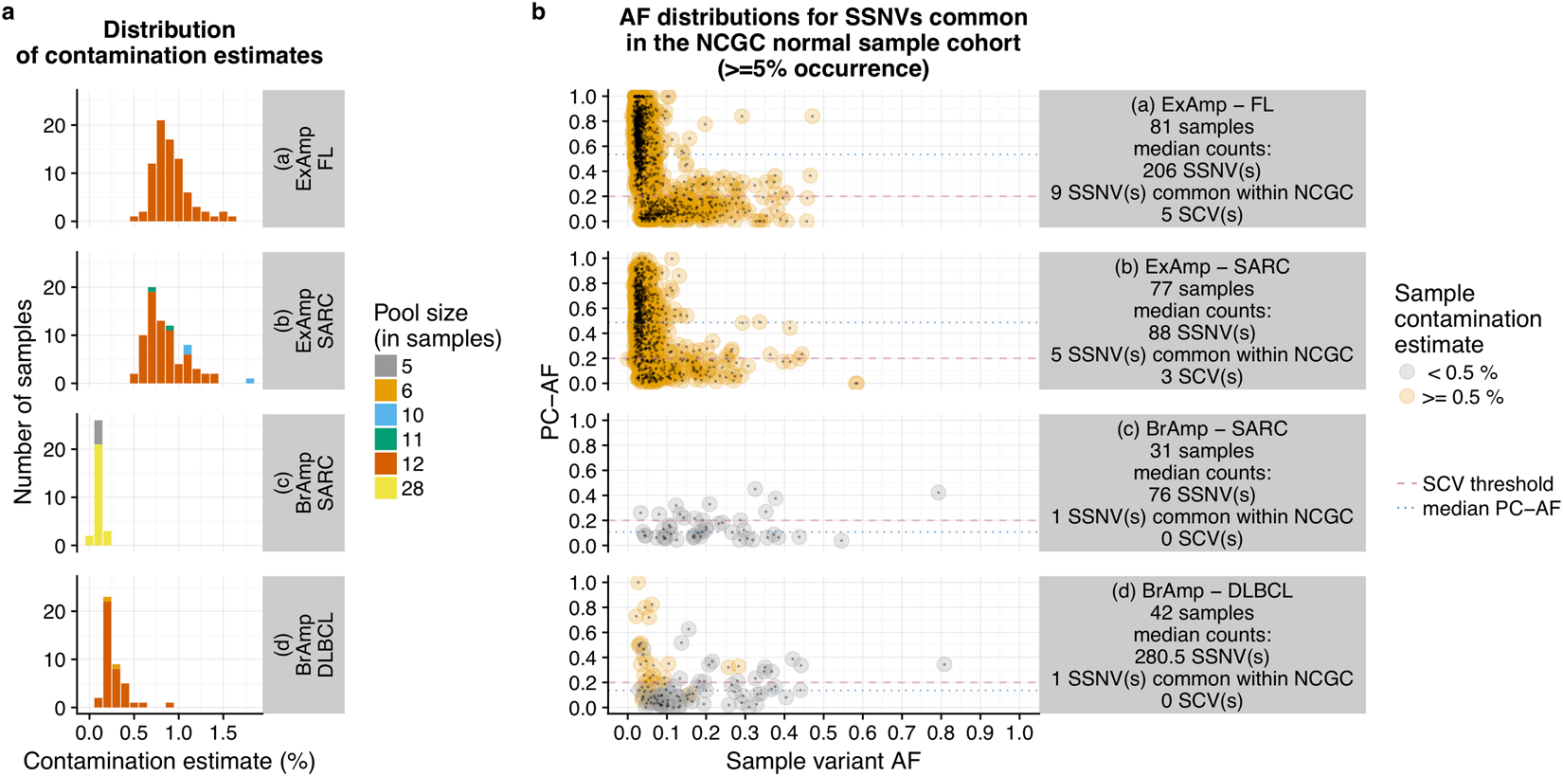
Amplification chemistry and its relationships to (**a**) sample contamination estimates and (**b**) variant counts and PC-AF values. All values are plotted separately for each combination of tumour type and amplification chemistry represented in the analysis. The colours distinguish between variants called in “high-contamination” samples (Conpair contamination estimate >  = 0.5%) and variants coming from samples with “low contamination” (Conpair contamination estimate < 0.5%). BrAmp: bridge amplification; FL: follicular lymphoma; SARC: sarcoma; DLBCL: diffuse large B-cell lymphoma; SCV: suspected contaminant variant.

During the analysis of hundreds of sequenced tumour-normal matched pairs, we noted that data generated on the ExAmp instrument showed significantly higher sample-wise contamination levels in comparison to data from bridge amplification instruments (median per-sample contamination estimates: 0.839% vs. 0.187%, p-value < 0.001, Fig. 2a, Supplementary Fig. 2). To specifically assess the role of multiplexing on the ExAmp instrument, we sequenced 16 selected sample libraries both in pools and individually (i.e., one sample library per flow cell lane), observing significantly increased contamination rates in sequencing output from the multiplexed libraries (median per-sample contamination estimates: 0.644% vs. 0.0465%, p-value < 0.001, Supplementary Table 3). Removal of free adapters/primers prior to sequencing was in Illumina’s report identified as a key measure for mitigating the cross-contamination rates (https://www.illumina.com/science/education/minimizing-index-hopping.html), but for the 16 libraries included in our testing, performing a gel-based library purification step in combination with bead purification did not provide improvements over bead purification alone (median per-sample contamination estimates: 0.671% vs. 0.6115%, p-value = 0.159). In accordance with previous reports by Illumina and Sinha *et al*., we concluded that ExAmp chemistry is the cause of sample contamination. The dependency on sample pooling indicated that co-multiplexed samples serve as the contaminants.

### Artefactual variant calls

In order to assess the impact of ExAmp-associated index misassignment on the detection of SSNVs, we first set out to identify SSNVs that were likely to originate from contamination. For each somatic variant found in a given tumour sample, we quantified the variant support in the expected source of contamination - the corresponding “pool complement” consisting of reads from all co-multiplexed samples from other individuals. We thus calculated two allelic fractions for each somatic variant: the standard sample AF and an AF derived from the pool complement (PC-AF) (Supplementary Fig. 3, Supplementary Information). Two distinct classes of variants can be identified when variant AFs and PC-AFs are plotted against each other: (1) apparently true somatic variants, consisting of variants not present in the Norwegian population (i.e., absent from NCGC’s cohort of normal samples) and lacking support in their respective pool complements (PC-AF < 0.01); and (2) suspected contaminant variants, consisting of common Norwegian germline variants (>  = 5% allele frequency in NCGC’s cohort of normal samples) with a considerable support in their respective pool complements (PC-AF >  = 0.2) (Supplementary Fig. 4). We classify the remaining variants as ambiguous.

**Figure 3:**
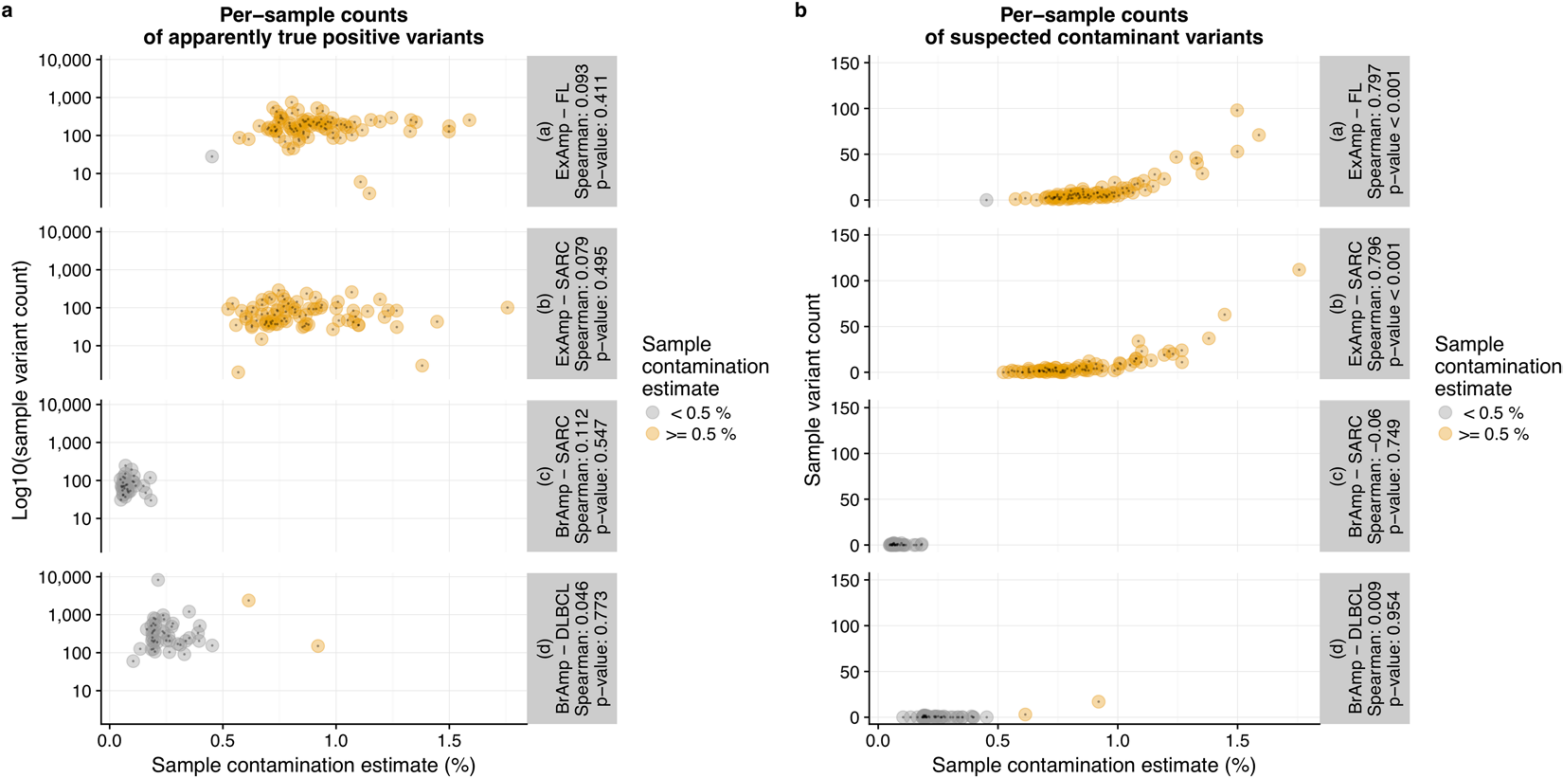
Per-sample counts of apparently true somatic variants (**a**) and suspected contaminant variants (**b**) plotted against contamination estimates. All values, including Spearman’s rank correlation coefficients and their associated p-values, are plotted separately for each combination of tumour type and amplification chemistry represented in the analysis. The colours distinguish between “high-contamination” samples (Conpair contamination estimate >  = 0.5%) and samples with “low contamination” (Conpair contamination estimate < 0.5%). BrAmp: bridge amplification; FL: follicular lymphoma; SARC: sarcoma; DLBCL: diffuse large B-cell lymphoma.

In comparison to bridge amplification, ExAmp leads to higher occurrence of SSNVs that coincide with germline variation common in the Norwegian population (Fig. 2b). At the same time, PC-AFs of these variants are significantly higher in the ExAmp datasets (median values: 0.508 vs. 0.125, p-value < 0.001), showing a much better correspondence to the suspected contamination source. Samples sequenced on the ExAmp instrument have significantly higher counts of suspected contaminant variants than samples sequenced on bridge amplification instruments (per-sample median counts: 4 vs. 0, p-value < 0.001) and show significant correspondence between the number of suspected contaminant variants and the estimated contamination (p-values < 0.001, Fig. 3).

## Discussion

We have focused on exploring the general link between tumour sample cross-contamination and suspected contaminant variants of germline origin, but the effects of index misassignment require further consideration (Table 1). Firstly, cross-sample contamination by recurrent somatic variants would represent a class of potential false positives that would be more difficult to recognize, and may have stronger clinical implications. Concern for such cases of contamination gains relevance when several samples containing identical somatic variants are being co-multiplexed and thereby jointly contribute to elevated pool complement support (e.g., pooling serial patient biopsies or screening multiple samples likely to harbour identical hotspot mutations, particularly in sensitive analyses of high-purity samples). Secondly, when co-multiplexing tumour and control samples, false negative variant rates might be increased due to somatic variation contaminating the controls. Lastly, contamination rates within a given sample can be influenced by a combination of independent factors, such as the fraction of the pool that is constituted by the pool complement^14^ or a sample’s copy number profile - in copy number loss regions of high-purity tumour samples, we expect both the average AF and the number of contaminant variants to be increased due to the local underrepresentation of non-contaminant reads.

**Table 1:** Overview of possible contamination types, their consequences and suitable filtering options. PC-AF: pool-complement allelic fraction.

Contamination type	Cause (the type of co-multiplexed samples)	Possible somatic variant calling artefacts	Prevalence of given contamination type in affected datasets	Suitable post-sequencing filtering options
a) Contaminant germline variants in a tumour sample	Any samples from other individuals	False positive somatic variants in the form of germline variation from other individuals	The most likely contamination type to occur; Contamination targets are expected to be more affected in copy number loss regions*	A variant filter based on an appropriate germline variant database or a relevant panel of normal samples; A filter based on PC-AF values (if a more discriminative solution is necessary)
b) Contaminant somatic variants in a tumour sample	Other tumour samples	False positive “recurrent” somatic variants in the form of somatic variation from other tumour samples – whether from other individual(s) or the same individual	Expected to be relevant in tumour sample pools enriched** for specific somatic variants; Contamination targets are expected to be more affected in copy number loss regions*	A filter based on PC-AF values (non-discriminative filtering might lead to false negatives of high importance)
c) Contaminant germline variants in a control sample	Any samples from other individuals	False negatives/missed somatic variant calls – only concerning somatic variants that also occur as germline variants	Dependent on the occurrence of important variants as both germline and somatic in a given project’s setting	Review of calls not classified as somatic, adjustment of the variant caller parameters
d) Contaminant somatic variants in a control sample	Any tumour samples	False negatives/missed somatic variant calls – concerning all somatic variants	Elevated relevancy when matched samples are co-multiplexed; Prevalence dependent on the enrichment** of potential contaminant variants in a given sample pool; Consequences dependent on variant caller’s tendency to reject a somatic variant candidate due to evidence of its presence in the matched control	Review of calls not classified as somatic, adjustment of the variant caller parameters

*Copy number loss regions of high-purity tumour samples will be especially affected.

**The enrichment will increase together with given variant’s recurrence, as well as with purity of tumour samples that carry the variant.

Several countermeasures have been suggested for the prevention of index misassignment, with sample storage conditions being shown by Illumina to influence the problem’s severity. Sequencing of a single sample per lane proved to be an effective solution, which however might be too costly for many applications. Rigorous gel- or bead-based library purification has been strongly recommended as a means of index misassignment mitigation in case of sample multiplexing. However, in our experience, both purification methods appear to be insufficient even in combination. Dual indexing has been suggested for circumventing the problem by using sample-specific index pairs rather than individual indexes, enabling recognition of reads with unexpected index combinations caused by index misassignment^14^. Dual indexes are becoming a part of best practices for multiplexed libraries sequenced with ExAmp chemistry, but their current availability may vary depending on the applied library preparation protocol. The possible solutions therefore need to be assessed in the context of each particular project.

For data that have already been generated, suitable variant post-processing should be chosen based on the type of contamination artefacts relevant for given project (Table 1). Commonly used allelic fraction thresholds and germline variant database filters are likely to remove the majority of false positive somatic variant calls caused by index misassignment. However, a more discriminative filtering approach would be preferable in settings where sensitive detection is the priority, such as in clonal analysis. Low-AF variation can, in multiple cancer types, harbour markers of prognostic and therapeutic value^15–17^. When available, pool complement information can help identifying variants that are unlikely to be contamination artefacts, thereby reducing the number of potentially important true positive somatic variants discarded due to low AF or germline database presence. The code used for PC-AF calculation in our analyses is available for use in other projects (Methods). If control samples have been contaminated by true somatic variation, it might be necessary to adopt variant caller settings more permissive to somatic allele evidence in the matched normal material.

We believe our findings to be of relevance to other cancer sequencing projects that utilize the ExAmp chemistry, even though the impacts of index misassignment on somatic variant calling may vary depending on the combination of employed sequencing instruments, library preparation protocols, and bioinformatics analyses. In general, we expect the effects of index misassignment to dampen as the sequencing depth decreases and the allelic fraction threshold for accepted somatic variants increases. On the other hand, we note that besides single nucleotide variants, other types of somatic variation (e.g. insertions and deletions) are likely to be affected.

## Methods

Methods used for sample collection, DNA extraction, library preparation and sequencing, as well as full details of all bioinformatics analyses, are available in the methods section of Supplementary Information.

### Bioinformatics analyses

All sequenced samples were pre-processed with BWA MEM^18^, Picard (http://broadinstitute.github.io/picard/) and GATK^19^ tools, before performing somatic calling with MuTect and Strelka (only consensus calls were considered in subsequent processing and testing). All analyses were performed using human reference genome build b37 with an added decoy contig and corresponding variation databases.

The pool complement of each individual tumour sample was formed by reads of all co-multiplexed samples originating from other individuals. For each identified somatic variant in a given tumour sample, all overlapping reads from the corresponding pool complement were extracted, and the pool complement allelic fraction (PC-AF, the fraction of reads supporting the variant within the pool complement) was calculated. Tumour samples without a matched control and/or tumour samples failing our quality metrics thresholds (Supplementary Information) were excluded from the analyses of potential contamination targets, but did serve as potential contamination sources in our PC-AF calculations.

For evaluating the role of multiplexing in ExAmp-associated index misassignment, tumour-normal sample pairs from 8 selected individuals were sequenced (i) individually (one sample per dedicated flow cell lane), (ii) in a pool of all 8 tumour/normal samples and (iii) in a pool of all 8 tumour/normal samples after applied gel purification. For generating contamination estimates with Conpair, the separately sequenced normal sample of each individual was used as a control for the five other samples of given individual.

Accuracy of Conpair contamination estimates was assessed with the help of artificially created exome-wide sample admixtures. Reads from multiple libraries were mixed to simulate contamination by 1, 2, 4 or 7 samples from as many different individuals, with the total contamination amounting to either ~2% or ~8% in each case, depending on the experiment. Conpair’s contamination estimates were compared to true contamination content, which was validated by library-specific depth of coverage calculations.

NCGC’s cohort of normal samples consisted of blood samples of 789 different individuals living in Norway. The samples were processed according to “Best Practices for Germline SNP & Indel Discovery in Whole Genome and Exome Sequence” developed by the Broad Institute, utilizing allele-specific (rather than site-specific) variant calling.

### Statistics

All statistical tests were performed in R (http:/www.r-project.org) as two-tailed non-parametric tests (either Wilcoxon rank sum test with continuity correction or Wilcoxon signed rank test) of the identity of two populations. All input test data are available as Supplementary Data. R session information, utilized R commands together with their outputs, as well as median and interquartile range values of all the tested distributions are available in the Supplementary Note. All distributions have been plotted as Supplementary Figures.

### Ethics approval and consent

All patients provided informed consent. The study was approved by the Regional Committee for Medical and Health Research Ethics of South-East Norway (reference numbers 2014/127, S-06133 and 2010/1244). All experiments were performed in accordance with relevant guidelines and regulations.

### Data availability

All data that support the findings of this study and that do not compromise research participant privacy are available as Supplementary Data. All data that may compromise research participant privacy (e.g., raw sequence data) are available for inspection upon request to the corresponding author [EH] on non-disclosure terms.

### Code availability

Python code used for calculating PC-AF values is available at GitHub (https://github.com/danielvo/IBPC). R code for generating Figures 2 and 3 is included in the Supplementary Note.

## Electronic supplementary material

Supplementary note

Supplementary Dataset Number 1

Supplementary Dataset Number 2

Supplementary Dataset Number 3

Supplementary Dataset Number 4

Supplementary Dataset Number 5

Supplementary Dataset Number 6
