# Supplementary material for: Sample-Index Misassignment Impacts Tumour Exome Sequencing
Source: Sci Rep. 2018 Mar 28;8:5307. doi: 10.1038/s41598-018-23563-4 (PMC5871786; doi:10.1038/s41598-018-23563-4)
Supplement: Supplementary file 1 — Supplementary note [file 41598_2018_23563_MOESM1_ESM.docx]

library(ggplot2)

library(cowplot)

library("plyr")

# a color blind friendly palette with grey, adapted from http://www.cookbook-r.com/Graphs/Colors_(ggplot2)/

cbPalette <- c("#999999", "#E69F00", "#56B4E9", "#009E73", "#D55E00", "#F0E442", "#0072B2", "#CC79A7")

## R session information

> sessionInfo()

# R version 3.4.1 (2017-06-30)

# Platform: x86_64-apple-darwin15.6.0 (64-bit)

# Running under: macOS High Sierra 10.13

#

# Matrix products: default

# BLAS: /Library/Frameworks/R.framework/Versions/3.4/Resources/lib/libRblas.0.dylib

# LAPACK: /Library/Frameworks/R.framework/Versions/3.4/Resources/lib/libRlapack.dylib

#

# locale:

# [1] C

#

# attached base packages:

# [1] stats graphics grDevices utils datasets methods base

#

# other attached packages:

# [1] plyr_1.8.4 cowplot_0.8.0 ggplot2_2.2.1

#

# loaded via a namespace (and not attached):

# [1] colorspace_1.3-2 scales_0.4.1 compiler_3.4.1 lazyeval_0.2.0 gtable_0.2.0

# [6] tibble_1.3.3 Rcpp_0.12.12 grid_3.4.1 rlang_0.1.1 munsell_0.4.3

## Test 1:

## Comparison of Conpair’s contamination estimates between

## tumor samples generated with exclusion amplification and

## tumor samples generated with bridge amplification.

vals_real <- read.table("Supplementary_Data_1.txt", header = T, sep="\t")

wilcox.test(CONPAIR_CONTAMINATION_ESTIMATE_TUMOR~Amplification, data = vals_real, conf.int = T)

## output:

# Wilcoxon rank sum test with continuity correction

#

# data: CONPAIR_CONTAMINATION_ESTIMATE_TUMOR by Amplification

# W = 114.5, p-value < 2.2e-16

# alternative hypothesis: true location shift is not equal to 0

# 95 percent confidence interval:

# -0.7130123 -0.6319833

# sample estimates:

# difference in location

# -0.670985

## creation of the associated supplementary figure

BrAmp_median <- round(median(vals_real[vals_real$Amplification == "BrAmp", ]$CONPAIR_CONTAMINATION_ESTIMATE_TUMOR), 3)

BrAmp_IQR <- round(IQR(vals_real[vals_real$Amplification == "BrAmp", ]$CONPAIR_CONTAMINATION_ESTIMATE_TUMOR), 3)

ExAmp_median <- round(median(vals_real[vals_real$Amplification == "ExAmp", ]$CONPAIR_CONTAMINATION_ESTIMATE_TUMOR), 3)

ExAmp_IQR <- round(IQR(vals_real[vals_real$Amplification == "ExAmp", ]$CONPAIR_CONTAMINATION_ESTIMATE_TUMOR), 3)

vals_real$AmplificationInfo <- "NA"

vals_real[vals_real$Amplification == "BrAmp", ]$AmplificationInfo <- paste("BrAmp-amplified samples", "\nmedian: ", BrAmp_median, "\ninterquartile range: ", BrAmp_IQR, sep = "")

vals_real[vals_real$Amplification == "ExAmp", ]$AmplificationInfo <- paste("ExAmp-amplified samples", "\nmedian: ", ExAmp_median, "\ninterquartile range: ", ExAmp_IQR, sep = "")

vals_real$contamination_class <- "NA"

vals_real[vals_real$CONPAIR_CONTAMINATION_ESTIMATE_TUMOR < 0.5,]$contamination_class <- " < 0.5 %"

vals_real[vals_real$CONPAIR_CONTAMINATION_ESTIMATE_TUMOR >= 0.5,]$contamination_class <- ">= 0.5 %"

plot_contam_all_sample <- ggplot(vals_real, aes(round(CONPAIR_CONTAMINATION_ESTIMATE_TUMOR, 1))) +

geom_bar(width=0.09, aes(fill=factor(contamination_class))) +

scale_fill_manual(values=cbPalette) +

facet_grid(AmplificationInfo~.) +

ylab("Number of samples") +

xlab("Sample contamination estimate (%)") +

ggtitle("Distributions of contamination estimates") +

background_grid(major = "xy", minor = "xy") +

guides(fill = guide_legend("Sample\ncontamination\nestimate")) +

theme(strip.text.y = element_text(angle = 90)) +

theme(panel.spacing = unit(1, "lines"))

save_plot("Supplementary_Figure_2.pdf", plot_contam_all_sample,

ncol = 2,

nrow = 1,

base_height = 6.5,

base_aspect_ratio = 1)

## Test 2:

## Comparison of SCV counts between

## tumor samples generated with exclusion amplification and

## tumor samples generated with bridge amplification.

vals_SCVs <- read.table("Supplementary_Data_2.txt", header = T, sep="\t")

wilcox.test(VARIANT_COUNT~Amplification, data = vals_SCVs, conf.int = T)

## output:

# Wilcoxon rank sum test with continuity correction

#

# data: VARIANT_COUNT by Amplification

# W = 1113.5, p-value < 2.2e-16

# alternative hypothesis: true location shift is not equal to 0

# 95 percent confidence interval:

# -4.999991 -2.999958

# sample estimates:

# difference in location

# -3.999954

## creation of the associated supplementary figure

BrAmp_median <- round(median(vals_SCVs[vals_SCVs$Amplification == "BrAmp", ]$VARIANT_COUNT), 3)

BrAmp_IQR <- round(IQR(vals_SCVs[vals_SCVs$Amplification == "BrAmp", ]$VARIANT_COUNT), 3)

ExAmp_median <- round(median(vals_SCVs[vals_SCVs$Amplification == "ExAmp", ]$VARIANT_COUNT), 3)

ExAmp_IQR <- round(IQR(vals_SCVs[vals_SCVs$Amplification == "ExAmp", ]$VARIANT_COUNT), 3)

vals_SCVs$AmplificationInfo <- "NA"

vals_SCVs[vals_SCVs$Amplification == "BrAmp", ]$AmplificationInfo <- paste("BrAmp-amplified samples", "\nmedian: ", BrAmp_median, "\ninterquartile range: ", BrAmp_IQR, sep = "")

vals_SCVs[vals_SCVs$Amplification == "ExAmp", ]$AmplificationInfo <- paste("ExAmp-amplified samples", "\nmedian: ", ExAmp_median, "\ninterquartile range: ", ExAmp_IQR, sep = "")

vals_SCVs$contamination_class <- "NA"

vals_SCVs[vals_SCVs$CONPAIR_CONTAMINATION_ESTIMATE_TUMOR < 0.5,]$contamination_class <- " < 0.5 %"

vals_SCVs[vals_SCVs$CONPAIR_CONTAMINATION_ESTIMATE_TUMOR >= 0.5,]$contamination_class <- ">= 0.5 %"

plot_contam_all_sample <- ggplot(vals_SCVs, aes(VARIANT_COUNT)) +

geom_bar(width=0.9, aes(fill=factor(contamination_class))) +

scale_fill_manual(values=cbPalette) +

facet_grid(AmplificationInfo~.) +

ylab("Number of samples") +

xlab("Number of suspected contaminant variants") +

ggtitle("Distributions of per-sample counts of suspected contaminant variant") +

background_grid(major = "xy", minor = "xy") +

guides(fill = guide_legend("Sample\ncontamination\nestimate")) +

theme(strip.text.y = element_text(angle = 90)) +

theme(panel.spacing = unit(1, "lines"))

save_plot("Supplementary_Figure_6.pdf", plot_contam_all_sample,

ncol = 2,

nrow = 1,

base_height = 6.5,

base_aspect_ratio = 1)

## Test 3:

## Comparison of PC-AF values between

## tumor samples generated with exclusion amplification and

## tumor samples generated with bridge amplification.

## Only PC-AF values for SSNVs common in the NCGC cohort of normals were included.

vars <- read.table("Supplementary_Data_3.txt", header = T, sep="\t")

wilcox.test(PC_AF~Amplification, data = vars, conf.int = T)

## output:

# Wilcoxon rank sum test with continuity correction

#

# data: PC_AF by Amplification

# W = 61187, p-value < 2.2e-16

# alternative hypothesis: true location shift is not equal to 0

# 95 percent confidence interval:

# -0.3710597 -0.2649853

# sample estimates:

# difference in location

# -0.3193147

## creation of the associated supplementary figure

BrAmp_median <- round(median(vars[vars$Amplification == "BrAmp", ]$PC_AF), 3)

BrAmp_IQR <- round(IQR(vars[vars$Amplification == "BrAmp", ]$PC_AF), 3)

ExAmp_median <- round(median(vars[vars$Amplification == "ExAmp", ]$PC_AF), 3)

ExAmp_IQR <- round(IQR(vars[vars$Amplification == "ExAmp", ]$PC_AF), 3)

vars$contamination_class <- "NA"

vars[vars$CONPAIR_CONTAMINATION_ESTIMATE_TUMOR < 0.5,]$contamination_class <- " < 0.5 %"

vars[vars$CONPAIR_CONTAMINATION_ESTIMATE_TUMOR >= 0.5,]$contamination_class <- ">= 0.5 %"

vars$AmplificationInfo <- "NA"

vars[vars$Amplification == "BrAmp", ]$AmplificationInfo <- paste("Variants from\nBrAmp-amplified samples", "\nmedian: ", BrAmp_median, "\ninterquartile range: ", BrAmp_IQR, sep = "")

vars[vars$Amplification == "ExAmp", ]$AmplificationInfo <- paste("Variants from\nExAmp-amplified samples", "\nmedian: ", ExAmp_median, "\ninterquartile range: ", ExAmp_IQR, sep = "")

plot_contam_all_sample <- ggplot(vars, aes(round(PC_AF, 1))) +

geom_bar(width=0.09, aes(fill=factor(contamination_class))) +

scale_fill_manual(values=cbPalette) +

facet_grid(AmplificationInfo~.) +

ylab("Number of variants") +

xlab("Variant PC-AF") +

ggtitle("Distributions of pool-complement allelic fraction (PC-AF) values\nfor SSNVs common in the NCGC cohort of normal samples") +

background_grid(major = "xy", minor = "xy") +

#guides(fill = guide_legend("Amplification\nmethod")) +

guides(fill = guide_legend("Sample\ncontamination\nestimate")) +

theme(strip.text.y = element_text(angle = 90)) +

theme(panel.spacing = unit(1, "lines"))

save_plot("Supplementary_Figure_7.pdf", plot_contam_all_sample,

ncol = 2,

nrow = 1,

base_height = 6.5,

base_aspect_ratio = 1)

## Test 4:

## Comparison of Conpair’s contamination estimates between

## samples sequenced in a pool and

## samples sequenced individually (one sample library per flow cell lane).

## Both tumor and normal samples were included in this test.

vals_ihop <- read.table("Supplementary_Data_4.txt", header = T, sep="\t")

wilcox.test(vals_ihop$alone, vals_ihop$pool, paired=TRUE, conf.int = T)

## output:

# Wilcoxon signed rank test

#

# data: vals_ihop$alone and vals_ihop$pool

# V = 0, p-value = 3.052e-05

# alternative hypothesis: true location shift is not equal to 0

# 95 percent confidence interval:

# -0.6615 -0.4625

# sample estimates:

# (pseudo)median

# -0.55025

wilcox.test(vals_ihop$pool, vals_ihop$pool_gel, paired=TRUE, conf.int = T)

## output:

# Wilcoxon signed rank test

#

# data: vals_ihop$pool and vals_ihop$pool_gel

# V = 40, p-value = 0.1591

# alternative hypothesis: true location shift is not equal to 0

# 95 percent confidence interval:

# -0.156 0.054

# sample estimates:

# (pseudo)median

# -0.05925

wilcox.test(vals_ihop$alone, vals_ihop$pool_gel, paired=TRUE, conf.int = T)

## output:

# Wilcoxon signed rank test with continuity correction

#

# data: vals_ihop$alone and vals_ihop$pool_gel

# V = 0, p-value = 0.0004814

# alternative hypothesis: true location shift is not equal to 0

# 95 percent confidence interval:

# -0.6859596 -0.5440582

# sample estimates:

# (pseudo)median

# -0.6137577

#

# Warning messages:

# 1: In wilcox.test.default(vals_ihop$alone, vals_ihop$pool_gel, paired = TRUE, :

# cannot compute exact p-value with ties

# 2: In wilcox.test.default(vals_ihop$alone, vals_ihop$pool_gel, paired = TRUE, :

# cannot compute exact confidence interval with ties

## code for generating Figure 1 from the Supplementary Data

vars <- read.table("Supplementary_Data_5.txt", header = T, sep="\t")

var_counts <- read.table("Supplementary_Data_6.txt", header = T, sep="\t")

vars_common <- vars[vars$NCGC_rarity == "Common",]

vars_common$contamination_class <- "NA"

vars_common[vars_common$CONPAIR_CONTAMINATION_ESTIMATE_TUMOR < 0.5,]$contamination_class <- " < 0.5 %"

vars_common[vars_common$CONPAIR_CONTAMINATION_ESTIMATE_TUMOR >= 0.5,]$contamination_class <- ">= 0.5 %"

vars_common$MEDIAN_PC_AF <- 0.0

vars_common[vars_common$Dataset == "A_ExAmp_FL",]$MEDIAN_PC_AF <- median(vars_common[vars_common$Dataset == "A_ExAmp_FL", c("PC_AF")])

vars_common[vars_common$Dataset == "B_ExAmp_SARC",]$MEDIAN_PC_AF <- median(vars_common[vars_common$Dataset == "B_ExAmp_SARC", c("PC_AF")])

vars_common[vars_common$Dataset == "C_BrAmp_SARC",]$MEDIAN_PC_AF <- median(vars_common[vars_common$Dataset == "C_BrAmp_SARC", c("PC_AF")])

vars_common[vars_common$Dataset == "D_BrAmp_DLBCL",]$MEDIAN_PC_AF <- median(vars_common[vars_common$Dataset == "D_BrAmp_DLBCL", c("PC_AF")])

vars_common$Dataset_info <- "NA"

vars_common[vars_common$Dataset == "A_ExAmp_FL",]$Dataset_info <- paste("A_ExAmp_FL\n",nrow(unique(vars[vars$Dataset == "A_ExAmp_FL", c("Dataset", "SAMPLE_ID")])), " samples\nmedian counts:\n", median(count(vars[vars$Dataset == "A_ExAmp_FL", ], "SAMPLE_ID")$freq), " SSNV(s)\n", median(var_counts[var_counts$Dataset == "A_ExAmp_FL" & var_counts$VARIANT_CLASS == "CNSSNV", ]$VARIANT_COUNT), " SSNV(s) common within NCGC\n", median(var_counts[var_counts$Dataset == "A_ExAmp_FL" & var_counts$VARIANT_CLASS == "SCV", ]$VARIANT_COUNT), " SCV(s)", sep = "")

vars_common[vars_common$Dataset == "B_ExAmp_SARC",]$Dataset_info <- paste("B_ExAmp_SARC\n",nrow(unique(vars[vars$Dataset == "B_ExAmp_SARC", c("Dataset", "SAMPLE_ID")])), " samples\nmedian counts:\n", median(count(vars[vars$Dataset == "B_ExAmp_SARC", ], "SAMPLE_ID")$freq), " SSNV(s)\n", median(var_counts[var_counts$Dataset == "B_ExAmp_SARC" & var_counts$VARIANT_CLASS == "CNSSNV", ]$VARIANT_COUNT), " SSNV(s) common within NCGC\n", median(var_counts[var_counts$Dataset == "B_ExAmp_SARC" & var_counts$VARIANT_CLASS == "SCV", ]$VARIANT_COUNT), " SCV(s)", sep = "")

vars_common[vars_common$Dataset == "C_BrAmp_SARC",]$Dataset_info <- paste("C_BrAmp_SARC\n",nrow(unique(vars[vars$Dataset == "C_BrAmp_SARC", c("Dataset", "SAMPLE_ID")])), " samples\nmedian counts:\n", median(count(vars[vars$Dataset == "C_BrAmp_SARC", ], "SAMPLE_ID")$freq), " SSNV(s)\n", median(var_counts[var_counts$Dataset == "C_BrAmp_SARC" & var_counts$VARIANT_CLASS == "CNSSNV", ]$VARIANT_COUNT), " SSNV(s) common within NCGC\n", median(var_counts[var_counts$Dataset == "C_BrAmp_SARC" & var_counts$VARIANT_CLASS == "SCV", ]$VARIANT_COUNT), " SCV(s)", sep = "")

vars_common[vars_common$Dataset == "D_BrAmp_DLBCL",]$Dataset_info <- paste("D_BrAmp_DLBCL\n",nrow(unique(vars[vars$Dataset == "D_BrAmp_DLBCL", c("Dataset", "SAMPLE_ID")])), " samples\nmedian counts:\n", median(count(vars[vars$Dataset == "D_BrAmp_DLBCL", ], "SAMPLE_ID")$freq), " SSNV(s)\n", median(var_counts[var_counts$Dataset == "D_BrAmp_DLBCL" & var_counts$VARIANT_CLASS == "CNSSNV", ]$VARIANT_COUNT), " SSNV(s) common within NCGC\n", median(var_counts[var_counts$Dataset == "D_BrAmp_DLBCL" & var_counts$VARIANT_CLASS == "SCV", ]$VARIANT_COUNT), " SCV(s)", sep = "")

vars_common$Dataset_info <- gsub("Amp_", "Amp - ", vars_common$Dataset_info)

vars_common$Dataset_info <- gsub("A_", "(a) ", vars_common$Dataset_info)

vars_common$Dataset_info <- gsub("B_", "(b) ", vars_common$Dataset_info)

vars_common$Dataset_info <- gsub("C_", "(c) ", vars_common$Dataset_info)

vars_common$Dataset_info <- gsub("D_", "(d) ", vars_common$Dataset_info)

AF_AF_plot <- ggplot(vars_common, aes(SAMPLE_VARIANT_AF, PC_AF)) +

geom_point(aes(col = contamination_class), size=3.75, alpha=0.25) +

scale_color_manual(values=cbPalette) +

geom_point(col="black", size=0.2, alpha = 0.25) +

coord_cartesian(xlim=c(0,1), ylim=c(0,1.19)) +

facet_grid(Dataset_info~.) +

ylab("PC-AF") +

xlab("Sample variant AF") +

ggtitle(paste("AF distributions for SSNVs common\nin the NCGC normal sample cohort\n(>=5% occurrence)", sep="")) +

scale_y_continuous(breaks = 0.2*c(0:5)) +

scale_x_continuous(breaks = 0.1*c(0:10)) +

coord_cartesian(xlim=c(0,1), ylim=c(0,1)) +

geom_hline(aes(linetype = "median PC-AF", yintercept = MEDIAN_PC_AF), data = vars_common, alpha = 0.5, col = "#0072B2") +

geom_hline(aes(linetype = "SCV threshold", yintercept = 0.2), alpha = 0.5, col = "#CC79A7") +

background_grid(major = "xy", minor = "xy") +

guides(col = guide_legend("Sample\ncontamination\nestimate")) +

scale_linetype_manual(name = "", labels = c("SCV threshold", "median PC-AF"), values = c("dashed", "dotted"), guide = guide_legend(override.aes = list(color = c("#CC79A7", "#0072B2")))) +

theme(strip.text.y = element_text(angle = 0)) +

theme(panel.spacing = unit(1, "lines"))

contamination_values <- unique(vars[, c("Dataset", "Amplification", "CONPAIR_CONTAMINATION_ESTIMATE_TUMOR", "SAMPLE_ID", "POOL_SIZE")])

contamination_values$Dataset <- gsub("Amp_", "Amp - ", contamination_values$Dataset)

contamination_values$Dataset <- gsub("A_", "(a)\n", contamination_values$Dataset)

contamination_values$Dataset <- gsub("B_", "(b)\n", contamination_values$Dataset)

contamination_values$Dataset <- gsub("C_", "(c)\n", contamination_values$Dataset)

contamination_values$Dataset <- gsub("D_", "(d)\n", contamination_values$Dataset)

contamination_values$Dataset <- gsub(" - ", "\n", contamination_values$Dataset)

cont_dist_plot <- ggplot(contamination_values, aes(round(CONPAIR_CONTAMINATION_ESTIMATE_TUMOR, 1))) +

geom_bar(width=0.09, aes(fill=factor(POOL_SIZE))) +

facet_grid(Dataset~.) + ylab("Number of samples") +

xlab("Contamination estimate (%)") +

ggtitle("Distribution\nof contamination estimates") +

background_grid(major = "xy", minor = "xy") +

scale_fill_manual(values=cbPalette) +

guides(fill = guide_legend("Pool size\n(in samples)")) +

theme(strip.text.y = element_text(angle = 90)) +

theme(panel.spacing = unit(1, "lines"))

plot_object <- plot_grid(cont_dist_plot, AF_AF_plot, labels = c("a", "b"), align = "h", rel_widths = c(1,2))

save_plot("Figure_1.pdf", plot_object,

ncol = 2,

nrow = 1,

base_height = 6.5,

base_aspect_ratio = 1)

## code for generating Figure 2 from the Supplementary Data

var_classes <- read.table("Supplementary_Data_6.txt", header = T, sep="\t")

corr_method <- "Spearman"

corr_method_par <- "spearman"

var_classes$Dataset_cor <- "Init"

for (dataset in c("A_ExAmp_FL", "B_ExAmp_SARC", "C_BrAmp_SARC", "D_BrAmp_DLBCL")) {

for (variation in c("SCV", "ATP")) {

row_index <- paste(dataset, "_", variation, sep = "")

data_subset <- var_classes[var_classes$VARIANT_CLASS == variation & var_classes$Dataset == dataset,]

data_test <- cor.test(data_subset$VARIANT_COUNT, data_subset$CONPAIR_CONTAMINATION_ESTIMATE_TUMOR, method = corr_method_par)

dataset_estimate <- round(data_test$estimate, 3)

dataset_p_value <- round(data_test$p.value, 3)

if (dataset_p_value > 0) {

dataset_p_value <- paste("p-value: ", dataset_p_value, sep = "")

} else {

dataset_p_value <- "p-value < 0.001"

}

var_classes[var_classes$VARIANT_CLASS == variation & var_classes$Dataset == dataset,]$Dataset_cor <- paste(dataset, "\n", corr_method, ": ", dataset_estimate, "\n", dataset_p_value, sep = "")

}

}

var_classes$Dataset_cor <- gsub("Amp_", "Amp - ", var_classes$Dataset_cor)

var_classes$Dataset_cor <- gsub("A_", "(a)\n", var_classes$Dataset_cor)

var_classes$Dataset_cor <- gsub("B_", "(b)\n", var_classes$Dataset_cor)

var_classes$Dataset_cor <- gsub("C_", "(c)\n", var_classes$Dataset_cor)

var_classes$Dataset_cor <- gsub("D_", "(d)\n", var_classes$Dataset_cor)

var_classes$contamination_class <- "NA"

var_classes[var_classes$CONPAIR_CONTAMINATION_ESTIMATE_TUMOR < 0.5,]$contamination_class <- " < 0.5 %"

var_classes[var_classes$CONPAIR_CONTAMINATION_ESTIMATE_TUMOR >= 0.5,]$contamination_class <- ">= 0.5 %"

var_classes_SCV_plot <- ggplot(var_classes[var_classes$VARIANT_CLASS == "SCV",], aes(CONPAIR_CONTAMINATION_ESTIMATE_TUMOR, VARIANT_COUNT)) +

geom_point(aes(col = contamination_class), size=3.75, alpha=0.4) +

scale_color_manual(values=cbPalette) +

geom_point(col="black", size=0.2, alpha = 0.25) +

facet_grid(Dataset_cor~.) +

scale_y_continuous("Sample variant count") +

xlab("Sample contamination estimate (%)") +

ggtitle(paste("Per-sample counts\nof suspected contaminant variants", sep="")) +

guides(col = guide_legend("Sample\ncontamination\nestimate")) +

background_grid(major = "xy", minor = "x") +

coord_cartesian(ylim=c(0, 150)) +

theme(strip.text.y = element_text(angle = 90)) +

theme(panel.spacing = unit(1, "lines"))

var_classes_ATP_plot <- ggplot(var_classes[var_classes$VARIANT_CLASS == "ATP",], aes(CONPAIR_CONTAMINATION_ESTIMATE_TUMOR, VARIANT_COUNT)) +

geom_point(aes(col = contamination_class), size=3.75, alpha=0.4) +

scale_color_manual(values=cbPalette) +

geom_point(col="black", size=0.2, alpha = 0.25) +

facet_grid(Dataset_cor~.) +

scale_y_log10("Log10(sample variant count)", breaks = c(1, 10, 100, 1000, 10000), labels=function(x) format(x, big.mark = ",", scientific = FALSE)) +

xlab("Sample contamination estimate (%)") +

ggtitle(paste("Per-sample counts\nof apparently true positive variants", sep="")) +

guides(col = guide_legend("Sample\ncontamination\nestimate")) +

background_grid(major = "xy", minor = "x") +

theme(strip.text.y = element_text(angle = 90)) +

theme(panel.spacing = unit(1, "lines"))

plot_object <- plot_grid(var_classes_ATP_plot, var_classes_SCV_plot, labels = c("a", "b"), align = "h")

save_plot(paste("Figure_2.pdf", sep = ""), plot_object,

ncol = 2,

nrow = 1,

base_height = 7.5,

base_aspect_ratio = 1)
